# The Relationship Between Serum Cytokine Levels and the Degree of Psychosis and Cognitive Impairment in Patients With Methamphetamine-Associated Psychosis in Chinese Patients

**DOI:** 10.3389/fpsyt.2020.594766

**Published:** 2020-12-11

**Authors:** Xue Yang, Hui Zhao, Xuebing Liu, Qin Xie, Xiaoliang Zhou, Qijian Deng, Gang Wang

**Affiliations:** ^1^Affiliated Wuhan Mental Health Center, Tongji Medical College of Huazhong, University of Science and Technology, Wuhan, China; ^2^First Affiliated Hospital of Xinjiang Medical University, Urumqi, China; ^3^Key Laboratory of Psychiatry and Mental Health of Hunan Province, China National Clinical Research Center for Mental Health Disorders, Mental Health Institute of the Second Xiangya Hospital, National Technology Institute of Psychiatry, Central South University, Changsha, China

**Keywords:** cognitive, methamphetamine, psychosis, serum cytokine, addiction

## Abstract

**Background:** Cytokine levels can be changed in methamphetamine (METH) use disorders (MUDs) and primary psychosis. The present study assessed serum levels of some kinds of interleukins (ILs) in METH-associated psychosis (MAP) and their relationships with psychotic symptoms and cognitive dysfunction.

**Methods:** Serum IL-2R, IL-6, IL-8, and IL-10 levels were examined by chemiluminescence assays in MAP patients (*n* = 119) and healthy controls (*n* = 108). The Positive and Negative Syndrome Scale (PANSS) and Montreal Cognitive Assessment (MOCA) were administered.

**Results:** Serum levels of IL-6 and IL-8 were significantly increased in MAP patients (all *p* < 0.05). There was a negative relationship between IL-2R levels and PANSS positive (P) subscale scores (*r* = −0.193, *p* = 0.035). IL-6, IL-8 and IL-10 levels were all negatively correlated with the naming, delayed recall and orientation subscores on the MOCA (*r* = −0.209, *p* = 0.022; *r* = −0.245, *p* = 0.007; *r* = −0.505, *p* < 0.001, respectively).

**Conclusions:** Our results indicate that immune disturbances are related to MAP and that IL-2R, IL-6, IL-8, and IL-10 are associated with the severity of psychotic symptoms and cognitive function impairment.

## Introduction

Methamphetamine (METH), a drug in the class of amphetamine-type stimulants (ATS), has been a global health issue for the past several decades, with an estimated 29 million people using ATS in 2017 (1). From 2010 to 2018, the number of registered ATS users has increased from 0.36 million to 1.35 in China (2). METH use places a heavy burden on families, society and the health care system.

Methamphetamine-associated psychosis (MAP) resulting from methamphetamine use, such as schizophrenia, depression, and other neuropsychiatric disorders (3–5). Moreover, patients with MUD have cognitive function deficits relative to healthy control subjects, including impairments of attention, executive function, and working memory (6). Most studies on MUDs have focused on neurotoxic effects, and the role of neuroinflammation in these toxic effects has been identified as important. METH exposure induces the release of a number of proinflammatory markers, including interleukin (IL)-1, IL-6, IL-8, and tumor necrosis factor-alpha (TNF-α), that cause neuroplasticity in the brain (7).

MUD also results in dysregulation in the peripheral immune system, leading to an imbalanced expression of cytokines. The expression of pro- and anti-inflammatory cytokines has been implicated in METH-related neuronal injury, which may be associated with MUD (8). Some studies have found that peripheral cytokines penetrate the blood-brain barrier directly via active transport mechanisms or indirectly via vagal nerve stimulation (9) and can relay messages responsible for inducing changes in motor function and motivation.

Many studies have examined the relationships between serum cytokine levels and the degree of psychosis in patients with primary mental disorders, such as schizophrenia and mood disorders (10, 11). However, studies examining MAP and cytokine levels are rare. The aim of our study was to examine the association of the intensity of psychiatric symptoms and the exacerbation of cognitive functions with imbalance in the immune system, which is reflected by serum cytokine concentrations, in MAP patients.

## Materials and Methods

### Subjects

Patients were recruited from Wuhan Mental Health Center (WMHC), a Wuhan city-owned psychiatric hospital. All patients met the following inclusion criteria: age 20–64 years and Han Chinese; confirmed diagnosis of MAP based on the Diagnostic and Statistical Manual of Mental Disorders 5 (DSM-5) by the consensus of two psychiatrists. A diagnosis of MAP was made if the following symptoms were present: (1) prominent hallucinations or delusions, a score ≥4 on at least one Positive and Negative Syndrome Scale (PANSS) (12) psychosis item (delusions, conceptual disorganization, hallucinatory behavior, grandiosity, or suspiciousness/persecution); (2) symptoms that met the criterion developed during or soon after substance intoxication or withdrawal or after exposure to METH, and the psychotic symptoms lasted for more than 2 weeks; (3) psychotic symptoms that were not part of a psychotic disorder (such as schizophrenia, schizophreniform disorder, or schizoaffective disorder, and if the psychotic symptom onset was prior to substance or medication use or persist longer than 1 month after substance intoxication or withdrawal, then another psychotic disorder is likely); (4) psychotic symptoms that do not only occur during delirium; and (5) use of METH at least once per week during the 3 months prior to enrollment and a positive urine screen for METH. The patients had not received any antipsychotic drugs, immunomodulators or antioxidants for at least 12 months before entry into the study.

Healthy participants were recruited in Wuhan by advertisements. None of them had taken any immunomodulators, antipsychotic drugs, or antioxidants during the 1 year before the study. None of the healthy subjects had a personal or family history of psychiatric disorder.

Subjects with primary psychosis, polydrug abuse or major medical conditions were excluded.

The study was approved by the WMHC Ethics Committee, and written informed consent was obtained from the participating patients and healthy individuals before enrollment, which is when the data were retrospectively collected.

### Clinical Measurements

The severity of psychotic symptoms was estimated with the PANSS. Cognitive functions were assessed using the Montreal Cognitive Assessment (MOCA) (13). Two psychiatrists who had simultaneously attended a training session before the use of the PANSS and MOCA administered these measures.

### Investigation of IL-2R, IL-6, IL-8, and IL-10 Levels in Serum

Blood samples were drawn from 7 to 9 a.m. after an overnight fast. The serum was separated by centrifugation and then stored at −70°C. Serum IL-2R, IL-6, IL-8, and IL-10 concentrations were analyzed with chemiluminescence assays. The reference ranges of IL-2R, IL-6, IL-8, and IL-10 were 223–710 U/mL, 0–5.9 pg/mL, 0–62 pg/mL, and 0–9.1 pg/mL, respectively.

Wuhan CMLabs is an independent clinical laboratory that holds a laboratory accreditation certificate from the China National Accreditation Service for Conformity Assessment (CNAS Number: MT0324), the College of American Pathologists certification (CAP Number: 8997670, AU-ID: 1734258) and biosafety laboratory certification, Hubei, China (BLS-2[2020]01-01-001).

### Statistical Analysis

Differences between patients and healthy controls were evaluated using independent *t*-tests for continuous variables and chi-square tests for demographic and clinical variables. Given that the IL data were not normally distributed in the two groups (Shapiro-Wilk test; IL-2R: *z* = 0.127, *p* = 0.000; IL-6: *z* = 0.327, *p* = 0.000; IL-8: *z* = 0.268, *p* = 0.000; IL-10: *z* = 0.352, *p* = 0.000 for patients; IL-2R: *z* = 0.933, *p* = 0.000; IL-6: *z* = 0.569, *p* = 0.000; IL-8: *z* = 0.819, *p* = 0.000; IL-10: *z* = 0.877, *p* = 0.000 for controls), a nonparametric test (Mann-Whitney test) was used for comparisons between the two groups.

We performed all correlation analyses using Pearson's correlation coefficient. We performed a multivariate regression analysis for each serum cytokine concentration, correcting for confounding variables, including age, education level and duration and frequency of METH use.

## Results

### Demographic Data

The study included 119 MAP inpatients with psychotic disorders, and the mean age of the patients was 34.5 ± 8.7 years. The mean age of the 108 controls was 35.3 ± 9.5 years. Age, gender, marital status and education did not differ significantly between the two groups; further details are shown in Table 1. There were insignificant correlations between gender, age and marital status, and serum IL-2R, IL-6, IL-8, and IL-10 levels are shown either for the whole group or for the two groups separately. However, the frequency of METH use and the time of onset psychotic symptoms after METH use were significantly correlated with IL-8 (*r* = 0.293, *n* = 119, *p* = 0.001, and *r* = 0.203, *n* = 119, *p* = 0.027).

**Table 1:** Demographics of patients and healthy control subjects.

	**Cases**	**Healthy**	***t* or χ^2^**	***p*-value**
	**(*n* = 119)**	**controls (108)**	**or *F***	
Gender			1.365	0.243
Male	102	98		
Female	17	10		
Marital status			0.154	0.985
Not married	56	52		
Married	49	42		
Divorced	12	12		
Widowed	2	2		
Age (years)	34.5 ± 8.7	35.7 ± 9.5	−0.956	0.34
Education (years)	11.0 ± 2.7	10.5 ± 2.8	1.155	0.25
Age of onset METH use (years)	25.7 ± 7.2			
Duration of METH use (years)	8.0 ± 4.1			
Frequency of METH use (times per week)	4.9 ± 5.1			
Time of onset psychotic symptoms after METH use (years)	3.9 ± 3.2			
PANSS total score	87.2 ± 18.7			
P subscore	25.5 ± 11.1			
N subscore	15.8 ± 9.5			
G subscore	46.7 ± 9.5			
MOCA total score	21.48 ± 2.49			
Visuospatial/executive subscore	3.43 ± 1.26			
Naming subscore	2.93 ± 0.28			
Attention subscore	1.29 ± 0.62			
Language subscore	1.18 ± 0.58			
Abstraction Subscore	1.38 ± 0.52			
Delayed recall subscore	2.20 ± 1.45			
Orientation subscore	5.92 ± 0.46			

*METH, Methamphetamine; PANSS, Positive and Negative Syndrome Scale; P, the positive scale; N, the negative scale; G, the general scale; MOCA, the Montreal Cognitive Assessment*.

### Serum Measures

Table 2 shows the comparison of the mean serum cytokine levels in MAP patients with those in normal controls.

**Table 2:** Multivariate regression analyses of the association between serum cytokine concentration and MAP, as measured in patients compared to healthy controls.

		**Standardized**		
		**regression coefficients**		
**Variables**		**Beta regression**		
		**coefficient**	***t***	***p*-value**
IL-2R	(Intercept)		5.461	0.000
	Gender	−0.128	−1.940	0.054
	Age	−0.038	−0.554	0.580
	Marital status	−0.074	−1.084	0.279
	Education	0.035	0.531	0.596
	Diagnosis	0.121	1.821	0.070
IL-6	(Intercept)		1.185	0.237
	Gender	−0.044	−0.668	0.505
	Age	−0.028	−0.409	0.683
	Marital status	0.081	1.192	0.235
	Education	0.104	1.560	0.120
	Diagnosis	−0.139	−2.096	0.037^*^
IL-8	(Intercept)		0.776	0.439
	Gender	−0.030	−0.461	0.645
	Age	0.027	0.397	0.692
	Marital status	0.110	1.636	0.103
	Education	0.133	2.013	0.045^*^
	Diagnosis	−0.174	−2.649	0.009^*^
IL-10	(Intercept)		3.652	0.000
	Gender	−0.055	−0.830	0.408
	Age	0.011	0.159	0.873
	Marital status	0.112	1.635	0.103
	Education	−0.130	−1.942	0.053
	Diagnosis	−0.009	−0.142	0.887

*METH, Methamphetamine*.

* *Statistically significant with p-value < 0.05*.

### Serum IL-2R Levels

The serum IL-2R levels were higher in the MAP patients {median [quartile range (QR)]: 338.0 (168.0)} than in the normal controls [median (QR): 380.5 (161.3); (*z* = −2.38; *p* = 0.017)]. In the multivariate regression analysis, however, this difference was not significant after adjusting for covariance in gender, age, marital status and education (*p* = 0.07) (Table 2).

### Serum IL-6 Levels

Serum IL-6 levels were higher in the MAP patients [median (QR): 3.8 (3.6)] than in the controls [median (QR): 3.3 (4.0); (*z* = −2.69; *p* = 0.007)]. In the multivariate regression analysis, this difference was still consistent after adjusting for covariation in age, gender, marital status and education (*p* = 0.037) (Table 2).

### Serum IL-8 Levels

Serum IL-8 levels were insignificantly different between the MAP group [median (QR): 163 (413)] and the control group [median (QR): 154.5 (164.8); (*z* = −0.852; *p* = 0.394)]. In the multivariate regression analysis, this difference was significant after gender, age, marital status and education were used as covariates (*p* = 0.009) (Table 2).

### Serum IL-10 Levels

Serum IL-10 levels were not significantly different between the MAP group [median (QR): 3.45 (1.85)] and the control group [median (QR): 3.47 (1.74); (*z* = −0.211; *p* = 0.833)]. In the multivariate regression analysis, this difference was still consistent after adjusting for covariation in gender, age, marital status and education (*p* = 0.887) (Table 2).

### The Relationship Among Cytokine Levels in Patients and Controls

**Figure 1:**
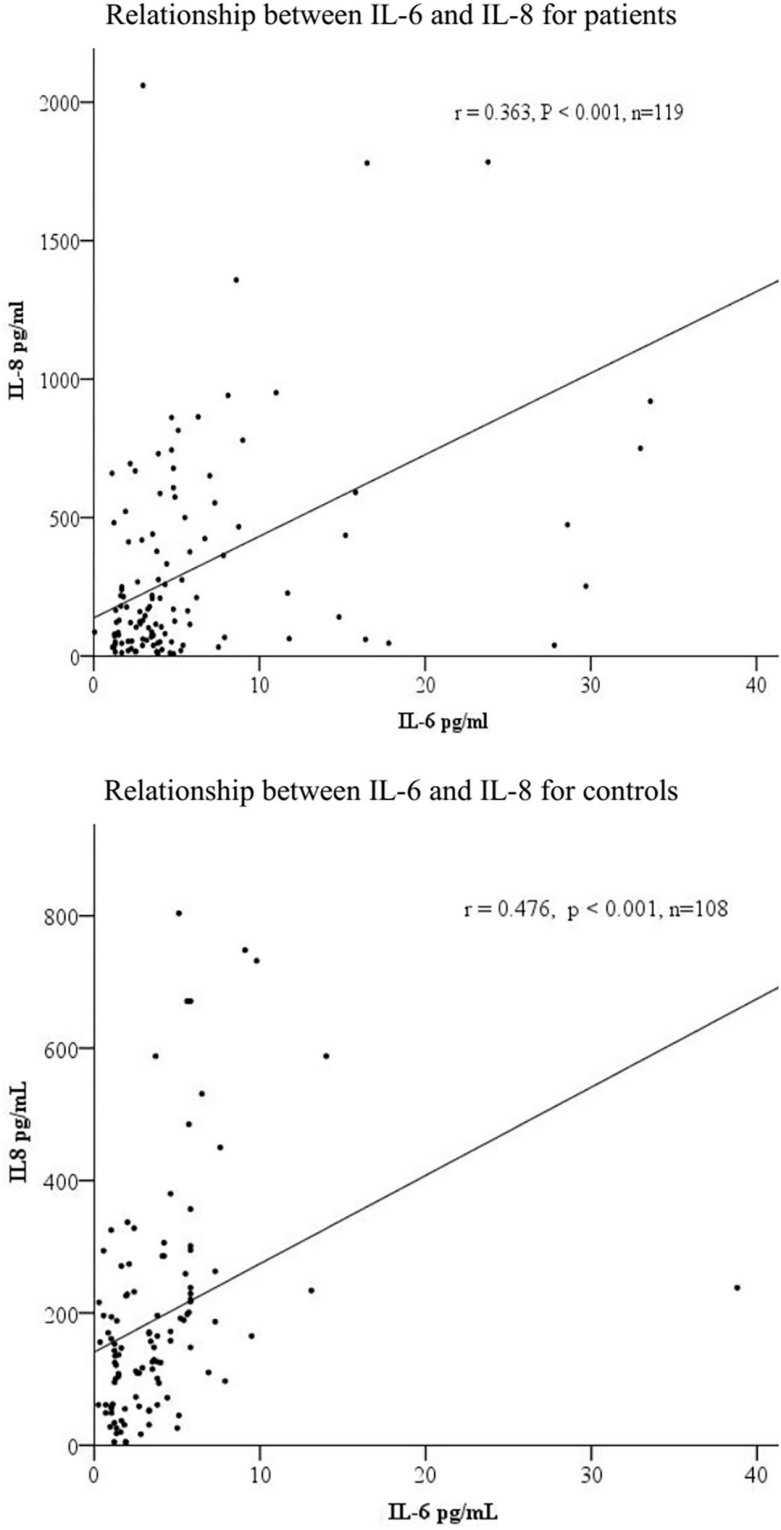
The correlations among serum IL-6 and IL-8 levels in patients (*n* = 119) and controls (*n* = 108).

Correlation analysis showed a significant and positive relationship between serum IL-6 and IL-8 levels in the MAP patients (*r* = 0.363, *p* < 0.001) and healthy controls (*r* = 0.476, *p* < 0.001) (Figure 1).

### The Relationship Between IL-2R, IL-6, IL-8, and IL-10 Levels and Clinical Symptoms

PANSS scores were found to have a statistically significant negative correlation with IL-2R levels (positive subscore: *r* = −0.193, *p* = 0.035). Correlations were found between IL-6, IL-8, and IL-10 levels and some clinical parameters, especially the severity of cognitive impairment. There was a significant negative relationship between serum IL-6 levels and MOCA scores (naming subscore: *r* = −0.209, *p* = 0.022). IL-8 levels were also found to have significant negative relationships with the delayed recall subscores on the MOCA (*r* = −0.245, *p* = 0.007), frequency of METH use (*r* = 0.293, *p* = 0.001), and time of psychotic symptom onset after METH use (*r* = 0.203, *p* = 0.027). There were significant and negative relationships between IL-10 and the orientation subscores on the MOCA (*r* = −0.505, *p* < 0.001). Further details are shown in Table 3.

**Table 3:** Pearson's correlations between clinical parameters and serum cytokine concentrations.

**Clinical**		**IL-2R**	**IL-6**	**IL-8**	**IL-10**
**parameters**					
P subscore	Correlation coefficient	−0.193^*^	0.062	0.081	−0.176
	*p*-value	0.035	0.505	0.382	0.056
N subscore	Correlation coefficient	−0.073	−0.047	−0.014	0.021
	*p*-value	0.430	0.613	0.878	0.825
G subscore	Correlation coefficient	−0.083	0.095	0.113	0.022
	*p*-value	0.367	0.302	0.221	0.813
Visuospatial/ executive subscore	Correlation coefficient	0.076	0.139	0.188^*^	0.130
	*p*-value	0.409	0.131	0.040	0.159
Naming subscore	Correlation coefficient	−0.009	−0.209^*^	−0.167	0.029
	*p*-value	0.923	0.022	0.069	0.751
Attention subscore	Correlation coefficient	−0.083	0.115	0.113	−0.031
	*p*-value	0.372	0.212	0.221	0.739
Language subscore	Correlation coefficient	−0.032	−0.008	−0.064	−0.024
	*p*-value	0.734	0.928	0.491	0.797
Abstraction subscore	Correlation coefficient	0.014	−0.053	−0.031	−0.062
	*p*-value	0.877	0.566	0.737	0.504
Delayed recall subscore	Correlation coefficient	−0.069	−0.045	−0.245^*^	0.134
	*p*-value	0.458	0.626	0.007	0.147
Orientation subscore	Correlation coefficient	−0.031	0.025	0.093	−0.505^*^
	*p*-value	0.742	0.789	0.317	0.000
Age of onset METH use	Correlation coefficient	−0.108	0.003	0.005	0.169
	*p*-value	0.243	0.974	0.956	0.067
Duration of METH use	Correlation coefficient	0.113	0.044	0.113	−0.112
	*p*-value	0.221	0.635	0.222	0.227
Frequency of METH use	Correlation coefficient	0.157	0.186^*^	0.293^*^	0.006
	*p*-value	0.087	0.043	0.001	0.948
Time of onset psychotic symptoms after METH use	Correlation coefficient	0.012	0.033	0.203^*^	−0.027
	*p*-value	0.894	0.719	0.027	0.767

*METH, Methamphetamine; PANSS, Positive and Negative Syndrome Scale; P, the positive scale; N, the negative scale; G, the general scale; MOCA, the Montreal Cognitive Assessment*.

* *Statistically significant with p-value < 0.05*.

### Cytokine Levels and the Severity of Clinical Symptoms

To further confirm the correlations between IL levels and the severity of psychotic symptoms, patients were placed in one of two subgroups based on their mean scores on the PANSS positive (*P*) subscale (25.5 ± 11.1), the negative (*N*) subscale (15.8 ± 9.5) and the general psychopathology (G) subscale (46.8 ± 9.5). When the patients were classified on the basis of their average PANSS *P* subscale scores, there was a trend toward lower IL-2R levels in the patients with high *P* subscale scores (*n* = 54) than in those with low *P* subscale scores (*n* = 65) (393.9 ± 150.8 vs. 333.6 ± 132.8 U/ml, respectively; *z* = −2.4, *p* = 0.017). IL-2R levels were lower in the patients with high G subscale scores (*n* = 47) than in those with low G subscale scores (*n* = 72) (399.7 ± 148.5 vs. 335.7 ± 135.9 U/ml, respectively; *z* = −2.4, *p* = 0.017).

To corroborate the correlations between IL levels and cognitive dysfunction, patients were placed in one of two subgroups based on the mean MOCA visuospatial/executive subscores (3.4 ± 1.3), naming subscores (2.9 ± 0.3), attention subscores (1.3 ± 0.6), language subscores (1.2 ± 0.6), abstraction subscores (1.4 ± 0.5), delayed recall subscores (2.2 ± 1.5), and orientation subscores (5.9 ± 0.5). Higher IL-8 levels were observed only in the patients with low delayed recall subscores (*n* = 70) compared with those with high delayed recall subscores (*n* = 49) (462.4 ± 656.1 vs. 165.2 ± 185.7 pg/ml, respectively; *z* = −3.4, *p* = 0.001).

## Discussion

This is the first study to evaluate the relationships between the severity of psychotic symptoms, cognitive abilities and serum levels of cytokines in MAP patients. The results of this study indicated that (1) serum levels of IL-6 and IL-8 were increased in MAP patients; (2) IL-2R levels had a negative correlation with the intensity of positive psychotic symptoms, but IL-6 and IL-8 levels did not; (3) cognitive dysfunction was present in the MAP patients (MOCA total scores of 26 and higher are generally considered normal); and (4) the severity of different types of cognitive impairment was positively correlated with IL-6, IL-8, and IL-10 levels. These results agree with the aforementioned findings indicating altered immune response activity in MAP patients (14–18).

The serum IL-2R levels in MAP patients were higher than normal in our study. Few studies have reported the relationship between IL-2R and MAP, but several studies have found that *in vivo* and *in vitro* exposure to METH resulted in changes in IL-2 levels. IL-2 plays a critical role in the immune response as a T-cell growth factor. Most studies have reported that serum IL-2 levels were reduced following METH exposure (18–20), which might result from decreased spleen, thymus, and peripheral T-lymphocyte cellularities (21). In METH-treated mice, there were decreases in the ratios of spleen and thymus weight to body weight, which indicated that METH induced immunosuppression and decreased IL-2 production by splenocytes (18). Moreover, IL-2 levels were lower in splenocytes from mice treated with concanavalin A (Con A) and METH than in splenocytes from mice treated with only Con A, which might be because *in vitro* METH exposure suppresses the induction of specific cytotoxic T-lymphocytes and alters the function of lymphocytes and NK cells (18, 19). Further study showed that exposure to METH induced mitochondrial dysfunction in the form of marked decreases in mitochondrial membrane potential, increased mitochondrial mass, enhanced protein nitrosylation and diminished protein levels of complexes I, III, and IV of the electron transport chain. These changes paralleled the reduction in IL-2 secretion and T cell proliferative responses after METH treatment (22). However, the finding of lower IL-2R levels in our present study was in conflict with the results of other studies (23, 24). This inconsistency may be due to the clinical status of the patients; for example, the participants in the Loftis study were adults in early recovery from METH dependence; in contrast, our participants were MAP inpatients, and their duration and frequency of METH use were different (23). In addition, the samples in some studies were brain tissue from mice, while our study used samples of peripheral serum in humans, which might have caused differences.

Another finding in this study was the significant negative relationship between IL-2R levels and PANSS positive subscale scores. These results suggest a possible correlation between IL-2R and the psychotic symptoms of MAP patients. There are few studies in this area, but the results of our present study agree with the findings in schizophrenia patients (25, 26). Studies have demonstrated that IL-2 increases dopamine (DA) turnover in the prefrontal cortex (27) and stimulates the release of DA in rat striatal cells (28). Higher CSF levels of IL-2 in neuroleptic-free schizophrenia patients could result in elevated DA neurotransmission and may contribute to psychotic symptoms (29). Moreover, IL-2 dose-dependently modulated the release of endogenous DA in a biphasic pattern, increasing release from striatal neurons at lower concentrations and inhibiting release at high concentrations, suggesting that the different IL-2 levels may play different roles in the pathophysiological process underlying psychotic symptoms (30).

We found that IL-6 and IL-8 levels were increased in MAP patients. Some studies have found that METH-activated microglia increase IL-6 and IL-8 expression in astrocytes (17, 31). mGluR5 receptor activity may be altered by METH, which activates the Akt/PI3K cascade. This may further activate the phosphorylation of IκB-α via the kinase activity of IKK, releasing the free form of heterodimeric NF-κB. Finally, active NF-κB translocates from the cytoplasm to the nucleus and promotes the transcription of IL-6 and IL-8 (17, 31). Another study showed that the PI3-Akt pathway and ERK1/2 mediated the induction of IL-6 and IL-8 by METH (15, 16). These findings were based on brain samples; however, METH has been shown to cause blood-brain barrier damage (32–34) and monocyte infiltration into the central nervous system (CNS) (35), and this, along with elevated levels of inflammatory markers, might indicate that peripheral cytokine levels are similar to CNS cytokine levels.

In our study, the interesting result was the consistent link between IL-6 and IL-8 levels and specific domains of cognitive performance. We found that increased IL-6 could be associated with naming impairments and that IL-8 could be associated with memory. Many studies have shown that abnormal IL-6 and IL-8 levels may be associated with cognitive dysfunction. Blood levels of IL-6 are significantly elevated in individuals with Alzheimer's disease (36, 37), and polymorphisms in the IL-6 gene that decrease plasma IL-6 levels may be associated with a lower risk of developing Alzheimer's disease (38). Increased serum concentrations of IL-6 and IL-8 have been associated with poor performance in memory, speed and motor function domains and predict increased risks of cognitive decline in an elderly general population (39). As levels of IL-8 increased, African Americans demonstrated slower reaction times on a delayed memory task (40). In addition to their roles in neurodegenerative processes, apoptosis and excitotoxicity, cytokines are capable of influencing neurotransmitter (41) and neuroendocrine responses (42) that subserve cognition and can directly modulate neuronal and glial cell function (43). These results indicate that the levels of peripheral IL-6 and IL-8 might be important biological indicators associated with the severity of cognitive dysfunction. It might be helpful to evaluate the severity of psychosis and develop a therapeutic strategy for MAP.

There were some limitations in this study. The elevated cytokine levels in MAP could easily be attributed to the psychotic condition instead of the MUD. We will design 3 groups, MAP, MUD without psychosis and healthy, in the next study to address this problem. There are few articles about serum cytokine levels in MAP, and in some articles about patients with schizophrenia, the sample size may have been insufficient, which raises the risk of spurious findings when making multiple comparisons. Moreover, the ability to extrapolate to larger human populations may be important given differences in drug use patterns and aspects of immune function across different ethnic populations.

## Conclusions

This study showed that IL-6 and IL-2R levels were elevated in MAP patients. Positive psychotic symptoms were found to negatively correlate with IL-2R, while the frequency of METH use and cognitive function were correlated with IL-6 levels. Such results suggest that immune disturbance may be involved in MAP and may have a relationship with more severe psychopathology in MAP.

## Data Availability Statement

The raw data supporting the conclusions of this article will be made available by the authors, without undue reservation.

## Ethics Statement

The studies involving human participants were reviewed and approved by Wuhan Mental Health Center Ethics Committee. The patients/participants provided their written informed consent to participate in this study.

## Author Contributions

GW, QD, XY, and HZ were responsible for the study concept and design. XY and XL contributed to the acquisition of patient' clinical data. XZ and HZ performed the serum cytokine analysis. QX, HZ, and GW assisted with data analysis and interpretation of findings. XY drafted the manuscript. GW and QD provided critical revision of the manuscript for important intellectual content. All authors critically reviewed content and approved final version for publication.

## Conflict of Interest

The authors declare that the research was conducted in the absence of any commercial or financial relationships that could be construed as a potential conflict of interest.
